# Knowledge and Perceptions of Health Workers’ Training on Ethics, Confidentiality and Medico-Legal Issues

**DOI:** 10.4172/2155-9627.1000205

**Published:** 2015-01-05

**Authors:** Bernard Asamoah Barnie, Paa Kobina Forson, Mercy Naa Aduele Opare-Addo, John Appiah-Poku, Gyikua Plange Rhule, George Oduro, Yaw Adu-Sarkodie, Peter Donkor

**Affiliations:** Kwame Nkrumah University of Science and Technology, Kumasi, Ghana

**Keywords:** Health workers, Ethics, Confidentiality, Medicolegal issues, Knowledge and perception, Training

## Abstract

**Introduction:**

Health care delivery in recent times has become more complicated, as patients expect health personnel to not only provide professional services but be accountable as well. It is thus imperative that health personnel are aware of their responsibility to the patient and also sensitive to medico legal issues if quality health care is to be assured.

**Objective:**

The aim of the study was to assess the knowledge and perception of health care workers on their training in ethics, confidentiality and medico-legal issues. It was expected that the results would inform policy on the training of the health workers.

**Method:**

A cross-sectional survey was conducted among some categories of health workers (Doctors, Nurses and Health care assistants) at the Accident and Emergency directorate of Komfo Anokye Teaching Hospital, Ghana. A self-administered questionnaire was used to elicit information on ethics, confidentiality and medico- legal issues. Data collected was analyzed using SPSS version 16.

**Results:**

A total of 103 health care workers were enrolled on the study representing 96% response rate. The study revealed that 74% had knowledge on ethics, confidentiality and medico- legal concepts; and 35.4% of the respondents indicated that health workers attitudes to ethics, confidentiality and medico- legal concepts was inadequate. About 28.3% indicated that their attitudes were good while 26.3% indicated attitudes were adequate with only 2% indicating that attitudes were very good. Nearly, 49% of the respondents also indicated that training on medico-legal issues should be taught during formal training and also on-the-job.

**Conclusion:**

Knowledge of health workers on ethics confidentiality and medico-legal issues is high and their perceptions are positive. However, regular training to update their knowledge will be necessary in order to ensure continuous improvement of the quality of health care delivery.

## Introduction

Global trends in medico-legal issues are gradually catching the attention of the public and complaints against physicians seem to be escalating in developing countries [1,2]. This has brought to the fore the need for a high sense of professionalism among health care practitioners. This professionalism relies heavily on the depth of knowledge and application of medical ethics in the everyday practice of the health care practitioners.

Understanding the attitudes of health workers on issues regarding patient confidentiality is vital in improving quality of care of patients, patient’s confidence in the health delivery system, and ultimately health outcomes. According to Nash (2007), education of health workers during pre-vocational and vocational training about the nature and impact of medicolegal matters is important in improving the quality of care and thereby strengthening the trust patients have in them [3].

Limentani (1999) posits that ethics are a central element in the quality of clinical practice and the care of patients [4]. However, medical schools may not be able to devote enough time to teaching of ethics, confidentiality and medico-legal issues. The curriculum on medico-legal issues may not be adequate or practical enough to enable the medical student reliably address all ethical dilemmas likely to be faced in practice [5,6].

Contending with ethical dilemmas in clinical practice is a daily occurrence in almost all health institutions worldwide [7,8]. These situations result in unpleasant conflicts between health care practitioners and patients which sometimes end in legal suits and litigations. In a study assessing the knowledge and attitudes on medico-legal issues and ethics in Barbados, up to a third of doctors and a quarter of nurses had no knowledge of existing ethical committees in their health institutions [9]. Knowledge of medico-legal issues among doctors in Nigeria was found to be grossly inadequate [10]. In another study by Sulmasy it was observed that inadequate knowledge of ethics and medico-legal issues could hinder transfer of knowledge to junior officers [11]. The gap between the faculty members’ confidence and knowledge could interfere with their abilities to model and teach ethics to house officers [12]. In the same study, it was found that knowledge of ethical issues did not differ so much between faculty (53%) and house officers (50%).

In recent times in Ghana, matters relating to ethics and medicolegal issues have become topical and have featured in the media. A study concluded in Ghana revealed that there is no consensus among health care practitioners on confidentiality matters regarding the management of HIV/AIDS [13]. This lack of consensus exposes the practitioners to the risk of ethical breaches during their practice lifetime. The main objective of this study was to assess the knowledge and perceptions of health workers’ on ethics, confidentiality and medico-legal issues.

## Methods

A cross-sectional anonymous survey was carried out in April 2013 in the Accident & Emergency Department of the Komfo Anokye Teaching Hospital (KATH). This is the second largest hospital in Ghana. The hospital provides healthcare services to about 450,000 outpatients and 30,000 in-patients annually. The Accident and Emergency (A & E) Department serves all directorates in the hospital. A purposive sample was drawn from healthcare practitioners at A & E Department. Those surveyed included Doctors, Nurses, and Healthcare Assistants in the various units of the Department.

A self-administered questionnaire was developed. The questionnaire was structured under ethics, confidentiality and medicolegal issues. There were four parts to the questionnaire. Section A sought information on demographics such as gender, age, profession, and the respondents’ work experience. Section B enquired about knowledge/awareness of medicolegal issues in medicine, Section C also looked at confidentiality and Section D focused on ethical issues.

Morgan’s table was used in determining the sample size. A sample of 103 health workers was considered desirable for the study. In order to determine the effectiveness of the research instrument, the questionnaire was pilot tested on ten health workers and modified accordingly. The researcher visited all the units of Accident & Emergency Department and personally handed over the questionnaire to 103 health workers. The completed questionnaires were collected after three weeks.

## Data Analysis

Data retrieved from respondents were entered into the SPSS 16 version statistical computer software and analysed using its data analysis programmes.

## Ethical Considerations

Ethical approval was sought from the Committee on Human Research Publications and Ethics of the School of Medical Sciences of the Kwame Nkrumah University of Science and Technology, Kumasi. Participants who consented to partake in the study were made to sign appropriate forms.

## Results

Ninety-nine questionnaires were retrieved giving a response rate of 96.1%. Of the 99 respondents, 58 (58.6%) were female and 41 (41.4%) were male. The respondents included medical staff (31.3%) and nursing staff (68.7%). Nearly 60% of the respondents were in the age group of 20-29 years. Thirty-six (36.4%) respondents were in the age group of 30-39 years, while only two (2.0%) were in the 40-49 year age group (Table 1). The majority 52 (52.5%) of the respondents had worked for between two to five years; 25.3% had worked for 1 year or less; and 21.2% had worked for 6 years or more.

To ascertain the level of knowledge of respondents on medico-legal issues, they were asked whether they had had any previous training.

Majority 53.5% had no formal training and 42.4% respondents had had some form of training. Fifty-two (52.5%) respondents found the pre-employment orientation course on medicolegal issues given to them as inadequate while 39 (39.4%) respondents found it adequate.

However, when respondents who had had training and those who found the orientation sessions adequate were cross tabulated, it was revealed that majority (31) of the respondents who had no training did not also find orientation course adequate while 22 respondents considered the orientation course as adequate (Table 2).

Regarding their source of knowledge it was observed that 29 (29.3%) respondents were taught ethics, confidentiality and medicolegal issues during their first year in their academic 4 studies while 19 (19.2%) had theirs during clinical rotations or during their housemanship. Thirty-four (34.3%) of the respondents wanted to receive training throughout the duration of their studies as well as on the job.

On respondents’ rating of health workers’ attitudes to ethics, confidentiality and medico-legal issues, 26 (26.3%) of the respondents rated health workers’ attitudes as adequate, 35 (35.4%) rated it inadequate, 28 (28.3%) rated it as good while 8 (8.1%) rated the attitude of health workers as very good. Two (2.0%) respondents did not answer. However, all except one (1.0%) respondent considered knowledge of medico-legal principles as important for providing quality health care to patients.

On issues relating to patient confidentiality, 63% participants answered the case scenarios correctly while 37% answered incorrectly. Participants were evenly split on issues surrounding revealing patient information to a third party payer (insurance provider). Eightyeight percent of participants correctly answered case scenarios concerning revealing patient information to a health care provider’s family.

Out of a set of four case scenarios regarding medical ethical issues, 88% (83) participants correctly answered the case scenarios while 12% answered incorrectly.

Respondents were asked to indicate whether they would like medico-legal issues to be taught differently to different categories of health workers. Nearly 49% of the respondents 5 confirmed the assertion that medico-legal issues be taught differently while 45.5% of the respondents thought otherwise. However, 6.1% of the respondents gave no answer.

## Discussion

The study revealed that all respondents were aware of ethics, confidentiality and medicolegal issues. Majority of respondents had had training during their first year in school even though they would have preferred training throughout their years of academic studies. This finding is consistent with findings by Hariharan et al. [9] that medical schools do not place a lot of emphasis on training on ethical and medico-legal issues. The call for training throughout the medical training years is in line with the recommendation for training on medico-legal issues in undergraduate and postgraduate levels [10]. The study reveals that, for 53% of participants orientation lectures on ethics and medico-legal issues were not adequate to equip them to face ethical challenges that they may come across.

Of concern is the 37% who answered incorrectly questions on patients confidentiality as it shows the need for further training of this group of practitioners. One striking observation in the study was the fact that almost all respondents agreed that knowledge of medico-legal principles was important in providing good healthcare to patients. This finding is consistent with a number of studies on ethical issues where participants clearly identified the link between quality health care provision and knowledge on ethics [14,15].

Out of a set of five case scenarios assessing the knowledge and awareness of medico-legal issues among health care providers, 73.6% of participants answered the case studies correctly whiles 24.4% answered incorrectly.

Majority (73.6%) of the health workers had fairly adequate knowledge about ethics, confidentiality and medico-legal issues. This differs from findings from an earlier study where knowledge scores were lower for faculty 53%; and house officers 50% [12].

Two-thirds (64%) of the respondents were aware of the need for confidentiality of patient information while 36% had no knowledge about these issues. All questions yielded a high percentage knowledge of more than 75% except the question on disclosure to a third party payor (insurance provider) where respondents were split in their decision. Comparing this with the study by Fadare et al. [10], this is high. However, both studies confirmed that knowledge of medicolegal issues among health workers is inadequate.

Nearly 49% of health workers recommended a change in how medico-legal issues are taught and suggested a) a problem oriented on-the-job training based of real situations; b) making it simple enough for all health workers to understand; and c) regular updates on new developments in the field.

## Conclusion

The knowledge of health workers on ethics, confidentiality and medico-legal issues is high. Health workers perception is positive. Even though healthcare practitioners may be fully aware of these issues and have fair knowledge, there is the need for them to receive further training regularly. Those who answered the scenarios wrongly could benefit from further on-the-job training on medico-legal issues. Healthcare practitioners need an inclusive and culturally relevant training based on practical situations to deepen their knowledge in order to ensure that patients have confidence in the health care system.

## Figures and Tables

**Table 1:** Distribution of respondents by Demographics

Characteristics	Values
Sex	
Male	41 (41.4%)
Female	58 (58.6%)
Profession	
Medical Staff	31 (31.3%)
Nursing Staff	68 68.7%)
Age	
Years 20 – 29	59 (59.6%)
Years 30 – 39	37 (37.4%)
Years 40 – 49	2 (2.0%)

**Table 2:** Cross tabulations between respondents who have had training and those who found the orientation course adequate.

Respondents who found the Orientation course adequate				Total
		Yes	No	42
Respondents who have had training	Yes	22	20	
	No	15	31	46
Total		37	51	88

**Table 3:** Confidentiality of patient information.

No.	Question Statement	True	False
1	Patient medical information may not be disclosed to anyone unless the individual signs a release of information form	77 (77.8%)	14 (14.1%)
2	Patient medical information may be disclosed to a third party pay or (insurance provider)	45 (45.5%)	43 (43.4%)
3	A big man in Kumasi is brought to the Accident and Emergency Centre with complications of HIV infection. You are allowed to tell your family at home about this big man’s diagnosis?	4 (4.0%)	87 (87.9%)
4	A big man in Kumasi is brought to the Accident and Emergency Centre with malaria. You are allowed to tell your family at home about this big man’s diagnosis.	8 (8.1%) 81	81.80%
